# Supplementary material for: Stability analysis of wheat lines with increased level of arabinoxylan
Source: PLoS One. 2020 May 8;15(5):e0232892. doi: 10.1371/journal.pone.0232892 (PMC7209258; doi:10.1371/journal.pone.0232892)
Supplement: S1 Table — (Martonvásár, 2013–2015 harvest years). (DOCX) [file pone.0232892.s001.docx]

S1 Table. Main growing conditions in different years of the experiment (Martonvásár, 2013-2015)

| **Termesztési körülmények** | | **2012/2013** | **2013/2014** | **2014/2015** |
| --- | --- | --- | --- | --- |
| **Location** | Geographic coordinates | 47.3N, 18.8E | | |
|  | Altitude | 115 m | | |
| **Growing parameters** | Previous crop | oilseed radish | oilseed radish | phacelia |
|  | Sowing density | 550 seeds/m^2^ | | |
| **Soil parameters** | Soil type | chernozem | | |
|  | PH (KCl) | 7.25 | | |
|  | Humus (m/m %) | 2.8 | | |
|  | P_2_O_5_ (mg/kg) | 210 | | |
|  | K_2_O (mg/kg) | 210 | | |
|  | Yearly average of N input through  NPK combined fertilizer  (active ingredient, kg/ha) | 120 | | |
