# Supplementary material for: Stability analysis of wheat lines with increased level of arabinoxylan
Source: PLoS One. 2020 May 8;15(5):e0232892. doi: 10.1371/journal.pone.0232892 (PMC7209258; doi:10.1371/journal.pone.0232892)
Supplement: S2 Table — (Martonvásár, 2013–2015 harvest years). (DOCX) [file pone.0232892.s002.docx]

S2 Table. Weather conditions in three seasons (2012-2015)

|  | **2012/2013** | **2013/2014** | **2014/2015** |
| --- | --- | --- | --- |
| Growing period in days | 273 | 268 | 276 |
| Cumulative Precipitation (mm) | 347 | 348,9 | 287,5 |
| Mean temperature © | 9,13 | 10,44 | 10,01 |
| Absolute min temp © | -13,00 | -11,80 | -15,40 |
| Absolute max temp © | 37,30 | 33,80 | 36,10 |
| Cumulative Precipitation (mm) in the last 100 days | 102,60 | 202,60 | 115,50 |
| Mean temperature © in the last 100 days | 19,08 | 18,33 | 18,80 |
| Absolute min temp © in the last 100 days | 4,90 | 2,60 | 0,20 |
| Absolute max temp © in the last 100 days | 37,30 | 33,80 | 36,10 |
| No of days with Tmin<=0 C | 93,00 | 71,00 | 82,00 |
| No of days with Tmin<=-10 C | 5,00 | 1,00 | 3,00 |
| No of days with Tmax>=25 C | 57,00 | 54,00 | 53,00 |
| No of days with Tmax>=30 C | 23,00 | 18,00 | 27,00 |
| No of days with Tmax>=35 C | 7,00 | 0,00 | 4,00 |
