# Supplementary material for: Stability analysis of wheat lines with increased level of arabinoxylan
Source: PLoS One. 2020 May 8;15(5):e0232892. doi: 10.1371/journal.pone.0232892 (PMC7209258; doi:10.1371/journal.pone.0232892)
Supplement: S3 Table — (DOCX) [file pone.0232892.s003.docx]

S3 Table. Correlations between the contents and quality and weather conditions in 31 wheat lines grown in three years

|  | *Cumulative Precipitation (mm)* | *Mean temperature ©* | *Absolute min temp ©* | *Absolute max temp ©* | *Cumulative Precipitation (mm) in the last 100 days* | *Mean temperature © in the last 100 days* | *Absolute min temp © in the last 100 days* | *Absolute max temp © in the last 100 days* | *No of days with Tmin<=0 C* | *No of days with Tmin<=-10 C* | *No of days with Tmax>=25 C* | *No of days with Tmax>=30 C* | *No of days with Tmax>=35 C* |
| --- | --- | --- | --- | --- | --- | --- | --- | --- | --- | --- | --- | --- | --- |
| Test weight | -0.597 | -0.691 | -0.811 | 0.908 | -0.978 | 0.893 | n.s. | 0.908 | 0.818 | 0.818 | n.s. | 0.932 | 0.863 |
| Thousand kernel weight | n.s. | -0.876 | -0.598 | 0.992 | -0.995 | 0.987 | n.s. | 0.992 | 0.953 | 0.953 | 0.477 | 0.780 | 0.975 |
| Flour yield | -0.994 | n.s. | -0.981 | n.s. | -0.518 | n.s. | -0.795 | n.s. | n.s. | n.s. | -0.586 | 0.902 | n.s. |
| Protein content | 0.995 | n.s. | 0.979 | n.s. | 0.510 | n.s. | 0.801 | n.s. | n.s. | n.s. | 0.594 | -0.897 | n.s. |
| Starch content | n.s. | -0.962 | -0.404 | 0.995 | -0.949 | 0.998 | 0.416 | 0.995 | 0.997 | 0.997 | 0.661 | 0.621 | 1.000 |
| Gluten content | -0.825 | -0.418 | -0.957 | 0.722 | -0.858 | 0.698 | -0.419 | 0.722 | 0.587 | 0.587 | n.s. | 0.999 | 0.651 |
| Gluten Index | 0.949 | -0.470 | 0.810 | n.s. | n.s. | n.s. | 0.976 | n.s. | n.s. | n.s. | 0.872 | -0.636 | n.s. |
| Zeleny sedimentation | 0.647 | -0.860 | 0.387 | 0.613 | -0.421 | 0.640 | 0.947 | 0.613 | 0.745 | 0.745 | 0.999 | n.s. | 0.687 |
| Farinograph Quality Number | -0.717 | -0.567 | -0.894 | 0.829 | -0.933 | 0.810 | n.s. | 0.829 | 0.716 | 0.716 | n.s. | 0.978 | 0.771 |
| Water absorption | 0.791 | -0.735 | 0.570 | 0.436 | n.s. | 0.467 | 0.993 | 0.436 | 0.590 | 0.590 | 0.985 | n.s. | 0.521 |
| Dough development time | -0.675 | -0.614 | -0.866 | 0.860 | -0.952 | 0.842 | n.s. | 0.860 | 0.755 | 0.755 | n.s. | 0.964 | 0.807 |
| Dough stability | -0.855 | -0.367 | -0.972 | 0.683 | -0.829 | 0.657 | -0.469 | 0.683 | 0.541 | 0.541 | n.s. | 1.000 | 0.609 |
| Dough softening at 10 min | 0.960 | n.s. | 1.000 | -0.472 | 0.657 | -0.442 | 0.679 | -0.472 | n.s. | n.s. | 0.438 | -0.962 | -0.385 |
| Dough softening at 12 min | 0.652 | 0.638 | 0.851 | -0.876 | 0.961 | -0.859 | n.s. | -0.876 | -0.775 | -0.775 | n.s. | -0.955 | -0.825 |
| TOT-pentosan | 0.964 | n.s. | 0.999 | -0.457 | 0.645 | -0.427 | 0.692 | -0.457 | n.s. | n.s. | 0.453 | -0.958 | -0.369 |
| WE-pentosan | 0.930 | n.s. | 0.998 | -0.550 | 0.723 | -0.521 | 0.610 | -0.550 | -0.392 | -0.392 | 0.355 | -0.983 | -0.466 |

n= 30, r _0.5%_=0.3494, r _0.1%_= 0.4093, r_0.01%_= 0.4487
