# Supplementary material for: Stability analysis of wheat lines with increased level of arabinoxylan
Source: PLoS One. 2020 May 8;15(5):e0232892. doi: 10.1371/journal.pone.0232892 (PMC7209258; doi:10.1371/journal.pone.0232892)
Supplement: Attachment — Submitted filename: Response to reviewers.docx [file pone.0232892.s004.docx]

**Response to reviewers**

Thank you for the reviewers for their work and valuable additions to the manuscript quality. Here are my answers to their comments bellow:

**Reviewer #1:**

The manuscript describes a study about a range of grain physical, chemical and quality traits of a suite of breeding lines via classic quantitative genetics approach. Based on my knowledge, the article is well written and contain some interesting discoveries. It involves a large amount of work which are professional designed and conducted. The manuscript is written in a clear and logical way, which is easy to understand. The conclusion well follows the results. In my view, this manuscript can be accepted for publishing on its current stand.

Thank you for the positive oppinion and the acceptance of the paper in its present form.

**Reviewer #2:**

The paper by Marianna et al. analyzed the stability of wheat lines with increased level of arabinoxylan in three years experiment. The author found that the contents of WE-fibre were highly heritable and wheat lines with high contents of WE-fibre also have good and stable processing properties. With no doubt, the research can arouse interests for the readers who engage in breeding wheat cultivars with the good quality and improved health benefits. However, the paper should add more detailed information in order to increase its theoretical component. The manuscript also has some grammar errors and inaccuracies should be revised. Some detailed suggestion for author as below:

1. Please describe the agricultural traits and quality traits of the 31 improved lines and 4 parents

Thank you for this note which was useful to consider. In the manuscript we referred to our previous paper (Tremme-Bede et al. 2017) several times in which we introduced the development of the lines studied in this paper and their most important agronomical and processing quality traits. As we introduced the most important traits of the lines there and do not want to fall into the problem of repetition, finally we decided to keep the original way and logic of the discussion, focusing on the most important traits being in relation to the arabinoxylan content. This is the reason for example that from the quality traits only the water absorption is discussed in more details.

2. The contents of protein and starch are low heritability in many reports. However, this research shows the heritability of the contents of protein and starch is very high (0.851 and 0.828, respectively). Why the results of this research are different from other reports, please discuss more about this.

Thank you. This is also an important note as well and I agree with that. The high effect of the genotype on the protein and starch content in this study might be resulted by fact that the studied lines went through several cycles of selection during the different generations and those lines were selected which not only have high AX, but also have high protein content and/or quality. This information or explanation is now added to the text.

3. In the methods part, please supply the methods for the test weight and thousand kernel weight.

The standard methods and the instruments used for the measurements were corrected and added.

4. The significant marker in Table1 should be * instead of X, please revise it .

Changes were made.

5. Line 89, there should be a full stop(.) after the ‘respectively’.

It was done so.

6. Line 347, ‘heritability values for TOT-AX then for WE-AX ‘ should be ‘heritability values for TOT-AX than for WE-AX ‘.

It was done so.

7. Line 349, ‘WE-AX then the G’ should be ‘WE-AX than the G’.

It was done so

**Journal Requirements:**

- Datas are available in a previous paper: Tremmel-Bede et al. 2017.
- Figures were checked with PACE.
- The manuscript was formatted to the PLOSOne style, both the title page and the main body of the paper.
- The text of the introduction and the methods were compared to the literature and the overlapping text were rephrased.
- Mentioning Figure 4 was deleted as it does not exist.
- Reference style and order were changed according to PLOSOne rules.
